# Covariation Between Palatal Shape and Craniofacial Morphology in Complete Unilateral Cleft Lip, Alveolus, and Palate: A Geometric Morphometric Study

**DOI:** 10.3390/jcm15186939

**Published:** 2026-09-08

**Authors:** Marek Skotarski, Andrzej Brudnicki, Anne Marie Kuijpers-Jagtman, Piotr S. Fudalej

**Affiliations:** 1 Department of Orthodontics, Jagiellonian University Medical College, Montelupich Str. 4, 30-155 Kraków, Poland; marek.skotarski@uj.edu.pl (M.S.); piotr.fudalej@uj.edu.pl (P.S.F.); 2 Department of Maxillofacial Surgery, Pediatric Surgery Clinic, Institute of Mother and Child, Kasprzaka Str. 17a, 01-211 Warsaw, Poland; andrzej.brudnicki@imid.med.pl; 3 Department of Orthodontics, University Medical Center Groningen, University of Groningen, 9713 GZ Groningen, The Netherlands; 4 Department of Orthodontics and Dentofacial Orthopedics, School of Dental Medicine, Medical Faculty, University of Bern, 3010 Bern, Switzerland; 5 Faculty of Dentistry, Universitas Indonesia, Campus Salemba, Jakarta 10430, Indonesia

**Keywords:** cleft lip and palate, geometric morphometrics, palatal morphology, craniofacial morphology, two-block partial least squares, shape covariation

## Abstract

**Background/Objectives**: Unilateral cleft lip, alveolus, and palate (UCLAP) is associated with disturbed maxillary and craniofacial growth, and palatal morphology may reflect congenital, growth-related, surgical, and orthodontic influences. This retrospective study used geometric morphometrics to investigate covariation between palatal and craniofacial morphology in children treated for complete UCLAP. **Methods**: Maxillary dental casts and lateral cephalometric radiographs of 48 non-syndromic patients (33 boys, 15 girls; mean age 9.9 years) were digitized using 239 palatal and 152 craniofacial landmarks and semilandmarks. Covariation was assessed by two-block partial least squares (PLS) analysis with permutation testing. **Results**: Overall covariation was not statistically significant (RV = 0.254, *p* = 0.097). PLS1 accounted for 28.9% of total squared covariance and had a block-score correlation of r = 0.658 (*p* = 0.084). Bootstrap resampling showed only partial reproducibility of the PLS1 directions. **Conclusions**: Palatal morphology does not appear to reliably reflect the overall craniofacial configuration in children with operated complete UCLAP. Further longitudinal studies using fully three-dimensional assessment are needed to clarify clinically meaningful palate–craniofacial relationships.

## 1. Introduction

Unilateral cleft lip, alveolus, and palate (UCLAP) is a complex craniofacial anomaly associated with disturbances in maxillary growth, occlusion, and overall craniofacial development. The phenotype observed in affected individuals reflects not only the congenital defect itself, but also the combined influence of growth-related and treatment-related factors that may further modify craniofacial morphology over time [1,2,3]. Management of complete UCLAP is multidisciplinary and staged. In this cohort protocol, two principal surgical stages preceded the study records: primary one-stage cleft repair and secondary alveolar bone grafting, followed by orthodontic treatment when indicated. Although these interventions are necessary to restore function and arch continuity, their timing and cumulative effects may also modify palatal and maxillary morphology, making it difficult to separate congenital variation from treatment-related change [2,3,4,5,6,7]. Among the structures affected in patients with clefts, the palate is of particular interest because it is directly involved in the congenital defect and is later modified by growth and treatment [2,4]. Postoperative scarring after palatoplasty is also relevant in this context, as it may adversely affect subsequent maxillary growth [3,5,6,7]. Palatal morphology may therefore represent not merely a local manifestation of the cleft, but also an imprint of the interaction between congenital, surgical, and orthodontic influences. Examining the relationship between palatal shape and craniofacial morphology may thus help to clarify to what extent cleft-related and treatment-related influences are reflected in the covariation between these two structures.

Previous studies have explored the relationship between palatal morphology and craniofacial form using conventional measurements rather than shape-based methods. In non-cleft subjects, such studies showed that maxillary arch dimensions and palatal height are associated with vertical facial morphology [8,9]. Forster et al. [10] and Grippaudo et al. [11] further reported that decreasing arch width was associated with an increasing angle between mandibular and cranial planes, while Ocak et al. [12] likewise found an association between narrower dental arches and a steeper mandibular plane. In cleft populations, analyses based on selected palatal dimensions and cephalometric variables indicated that palatal morphology may be influenced by factors such as cleft type, dentition stage, missing teeth, and certain skeletal relationships [13]. Other studies linked cleft-related palatal characteristics to subsequent maxillary growth, further supporting an association between palatal morphology and craniofacial development [14,15,16]. In particular, Peltomäki et al. [14] found that cleft width was related to maxillary length (PNS-ANS distance) and sagittal maxillary position relative to the cranial base (SNA, BaNA angles), while stronger associations were observed when cleft width was evaluated in proportion to arch circumference or arch length. In addition, Jonsson and Thilander reported that the extent of clefting was associated with differences in arch dimensions and craniofacial morphology, whereas occlusion did not appear to vary accordingly [17]. Together, these studies provided valuable information on palate–craniofacial relationships. However, because they relied on selected linear and angular variables, they may have captured these relationships only partially, particularly when the aim is to determine whether palatal variation reflects broader craniofacial morphology rather than isolated craniofacial measurements.

Geometric morphometric methods are well suited to assessing the relationship between palatal and craniofacial morphology, not only because they offer statistical rigor but also because they enable biologically meaningful visualization and quantification of complex morphological relationships [18,19,20]. This is possible because of Procrustes superimposition, which removes the effects of translation, rotation, and scale while treating all landmarks equally—unlike conventional superimposition methods, which rely on selected reference structures and therefore weight some landmarks more heavily than others [21]. In non-cleft populations, previous morphometric studies have demonstrated significant associations between palatal shape and craniofacial morphology, although these associations have generally been of moderate strength. These studies have consistently indicated that the vertical skeletal pattern is an important determinant of palatal form, with high-angle subjects tending to present a narrower and higher palate [22,23,24]. Ahn et al. further demonstrated, using geometric morphometrics and structural equation modeling, that palatal morphology is associated with the craniofacial skeletal pattern in Class III subjects, with the transverse skeletal dimension exerting the strongest influence on palatal shape [25]. Similarly, Laganà et al. showed that growing subjects with skeletal open bite presented a significantly narrower and higher palate than controls, further supporting the association between vertical skeletal pattern and palatal morphology [26]. Together, these findings support the view that the palate and the craniofacial complex represent interconnected morphological systems rather than independent anatomical regions.

In cleft populations, morphometric studies suggest that craniofacial morphology is more variable than in unaffected individuals. Geometric morphometric studies have shown broader craniofacial variability in operated unilateral cleft lip and palate patients than in unaffected individuals, whereas investigations of unrepaired unilateral clefts suggest that at least part of this variability may reflect intrinsic growth deficiency rather than solely secondary effects of surgical treatment [27,28]. However, cleft-related studies have so far focused mainly on craniofacial morphology alone [1,28] or on descriptive assessments of craniofacial and palatal morphology considered separately [29], rather than on direct quantification of covariation between palatal and craniofacial shape. Consequently, it remains unclear to what extent variation in palatal shape is associated with variation in craniofacial morphology in operated patients with complete UCLAP.

Therefore, the present study examined whether variation in palatal morphology is accompanied by corresponding variation in the craniofacial complex in children with complete UCLAP using geometric morphometric methods.

## 2. Materials and Methods

### 2.1. Study Design

This retrospective cross-sectional study was based on pre-existing clinical records, dental casts, and lateral cephalometric radiographs retrieved from the institutional archive. The study was approved by the Bioethics Committee of the Institute of Mother and Child in Warsaw, Poland (reference no. 21/2013, 16 October 2013). All procedures were performed in accordance with the Declaration of Helsinki and relevant institutional guidelines and regulations. Patient consent was waived owing to the use of retrospective, anonymized data.

### 2.2. Setting

The study was conducted at the Institute of Mother and Child in Warsaw, Poland. The archive search covered patients treated from 2016 to 2020. The dental casts and lateral cephalometric radiographs used for analysis were obtained between 2016 and 2020.

### 2.3. Participants

The study group included only patients with complete UCLAP who met the following inclusion criteria: completion of all cleft-related surgical procedures at the Institute of Mother and Child, and availability of good-quality dental casts and lateral cephalometric radiographs obtained within 12 months of each other. Eligibility was verified by clinicians from the medical records. Patients were excluded if they had syndromic clefts, incomplete records, inadequate dental casts or radiographs, or craniofacial anomalies other than cleft lip and palate. The presence of a Simonart’s band was not a reason for exclusion. Of the 48 included participants, 36 had a left-sided and 12 a right-sided complete UCLAP.

### 2.4. Treatment Protocol

All cleft-related surgical procedures, including early secondary alveolar bone grafting, were performed at the Institute of Mother and Child in Warsaw, Poland, by a team of five experienced cleft surgeons; primary one-stage UCLAP repairs were restricted to the three most experienced surgeons in the team. In this cohort, early secondary alveolar bone grafting was performed most often between 2 and 4 years of age by members of the same surgical team according to a standardized technique. A cortico-cancellous bone block harvested from the anterior iliac crest was inserted between the bony margins of the alveolar cleft with the cortical lamina directed labially, and residual spaces were filled with cancellous bone chips. Minimal elevation of the palatal periosteum was performed before the graft was covered with gingival mucoperiosteal flaps. No fixation devices were used. Any oronasal fistula present at the time of grafting was closed during the same procedure. The operative protocols for primary one-stage repair and alveolar bone grafting were standardized across surgeons.

Where orthodontic care was provided, it consisted chiefly of transverse maxillary expansion directed at posterior crossbites, usually begun before the age of 6 and delivered most often with removable Schwarz plates. Once the active phase was finished, the plate was retained to maintain the transverse correction. Quad-helix appliances were used occasionally, and fixed appliances were introduced only when alignment of the maxillary central incisors was required. Protraction face masks and skeletal anchorage were not used, and Hyrax-type appliances were not described in the documented routine protocol for this cohort. Detailed patient-level information on dentition stage at treatment and on the exact type, timing, duration, and extent of previous orthodontic treatment was not consistently available in the archival records. Consequently, these treatment variables could not be used for stratification or adjustment.

### 2.5. Baseline Cleft Severity

The diagnosis of complete UCLAP was documented in the clinical records. Quantitative information on baseline cleft severity, including the anteroposterior extent of the cleft and the transverse gap between the major and minor maxillary segments, was not available because standardized presurgical dental casts or three-dimensional scans had not been retained.

### 2.6. Variables

The primary relationship of interest was the covariation between palatal shape and craniofacial shape. Palatal and craniofacial shape were treated as two separate blocks of shape variables for geometric morphometric analysis. Palatal shape was represented by Procrustes-superimposed landmark coordinates obtained from three-dimensional palatal models, whereas craniofacial shape was represented by Procrustes-superimposed landmark coordinates obtained from lateral cephalometric radiographs. Age and sex were recorded as participant characteristics but were not entered as covariates in the primary two-block PLS analysis. Sex-related differences in palatal and craniofacial shape were assessed separately by permutation testing; because no statistically significant differences were detected, boys and girls were pooled for subsequent analyses. Diagnostic classification of complete UCLAP and non-syndromic status was based on information recorded in the medical records and verified by clinicians during participant selection.

### 2.7. Data Sources and Measurement

**Figure 1:**
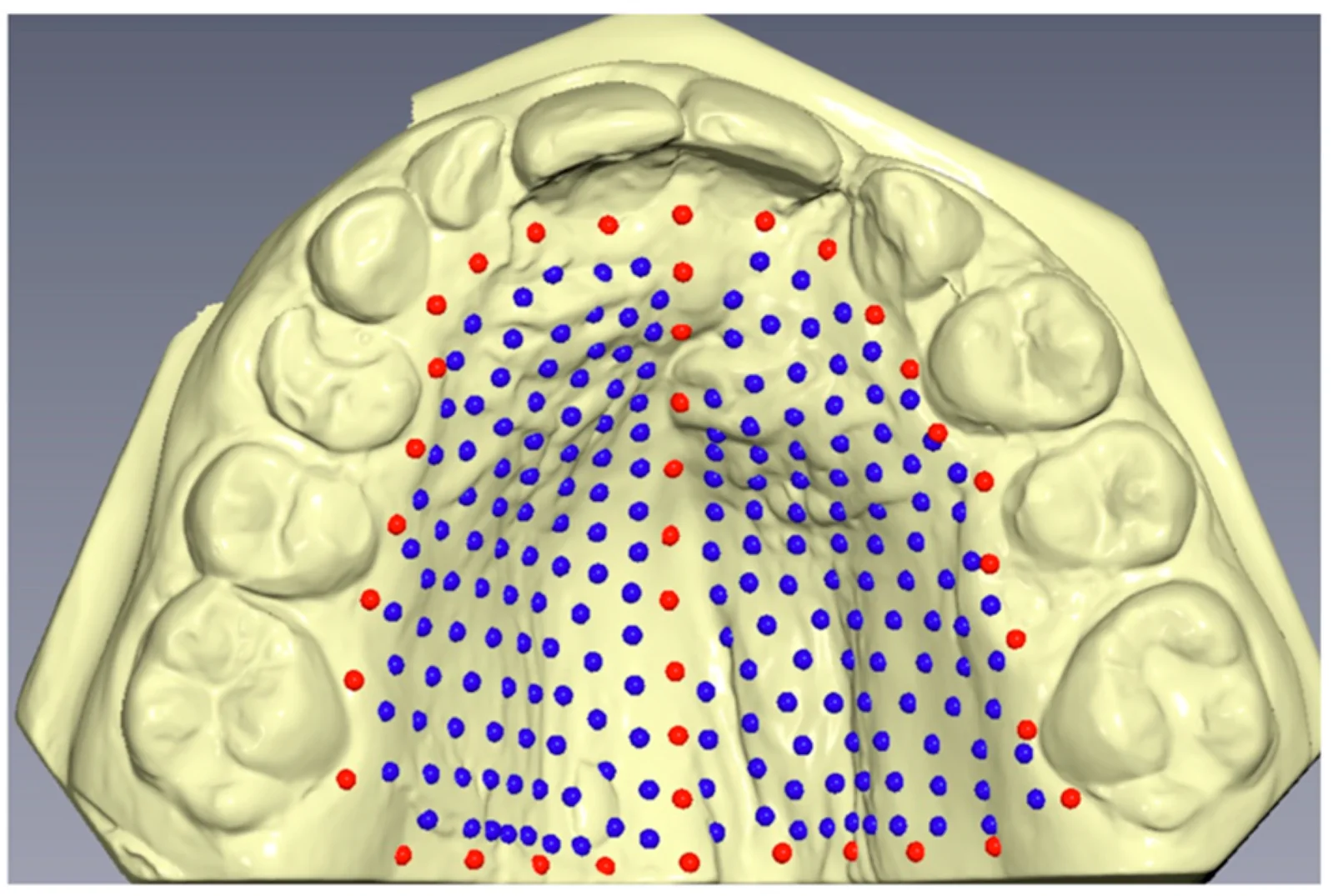
The curves traced on the digital casts. Red: midsagittal suture, dental arch outline along the margin, and posterior boundary tangent to the distal surface of the permanent first molars; blue: semilandmarks across the palatal surface. Reproduced from Petrova et al. (2023), J. Clin. Med. 12, 5985 [31], under the terms of the Creative Commons Attribution (CC BY) license; © 2023 the authors.

Palatal shape was assessed from conventional maxillary plaster (gypsum) casts. The physical casts were digitized using the TRIOS 3 intraoral scanner (3Shape A/S, Copenhagen, Denmark) and saved as STL files; no 3D-printed models were used. Use of a TRIOS scanner to digitize gypsum casts has previously been evaluated for orthodontic model analysis, with comparable conventional and digital arch measurements reported [30]. In cases where the cleft was situated on the right side, the digital scan was horizontally mirrored to ensure that the cleft was consistently on the left side for all subjects. A total of 239 landmarks were identified on each digital model using the Viewbox 4 program (dHAL Software v. 4, Kifissia, Greece). The palatal landmark configuration is illustrated in Figure 1 and follows the configuration previously published by Petrova et al. [31].

Initially, 39 of these landmarks, referred to as “fixed landmarks,” were manually placed on the palate. Among these fixed landmarks were 9 points representing the midsagittal line, 21 points outlining the dental arch and passing apically to the gingival sulci of each tooth, and 9 points defining a posterior curve that extended from the distal aspect of the first permanent molars, perpendicular to the midsagittal line (Figure 1). The remaining landmarks, designated as “semi-landmarks,” were automatically positioned uniformly on the palatal surface within the boundaries defined by the fixed landmarks. Subsequently, these semi-landmarks were adjusted to minimize bending energy, projected back onto the palatal surface, and further adjusted through sliding. This sliding-projecting process was iterated three times, ensuring the homologous positioning of all landmarks across subjects [32]. The palatal vault was assessed up to the gingival margin to eliminate the influence of dental inclination and tooth position on the alveolar bone [24]. All palatal models of patients with UCLAP were digitized and landmarked using the same protocol by a single examiner (MS).

**Figure 2:**
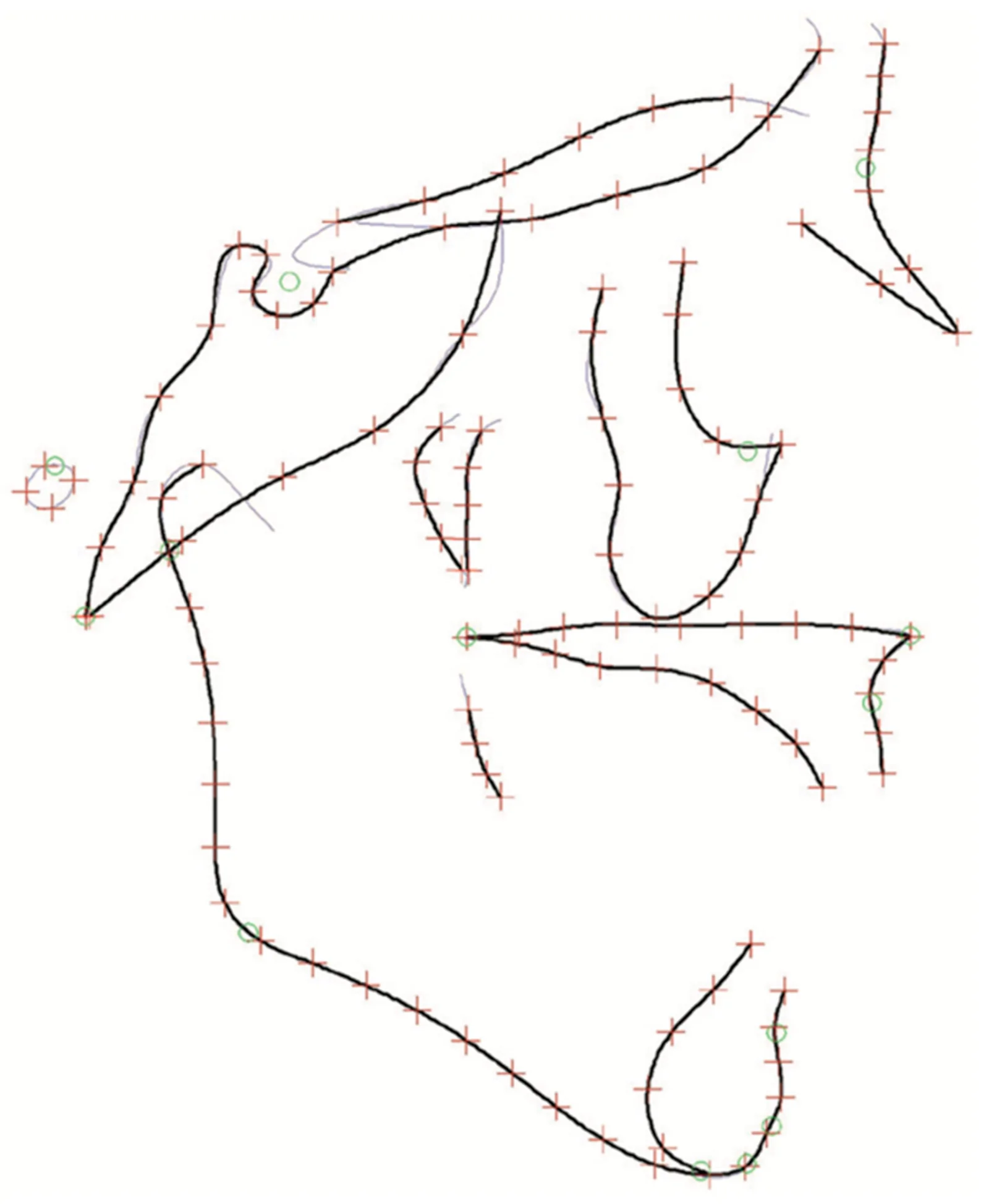
Anatomical landmarks (green circles) and sliding semilandmarks (red crosses) defining the craniofacial skeletal complex.

Craniofacial shape was assessed from lateral cephalometric radiographs. The craniofacial complex was digitized in Viewbox 4 (dHAL Software, Kifissia, Greece) according to a previously described geometric morphometric protocol, using a landmark and curve configuration similar to that in the study by Paoloni et al. [23]. In total, 152 points were used to describe craniofacial shape, comprising 14 fixed cephalometric landmarks and 138 semilandmarks placed at equal intervals along 15 continuous curves [23] (Figure 2). All tracings were performed by a single examiner (MS). After calculation of the Procrustes mean configuration, semilandmarks were allowed to slide along the curves to reduce thin-plate spline (TPS) bending energy and to improve homology across subjects. The sliding procedure was repeated twice. Landmark identification was standardized using predefined geometric morphometric protocols: the palatal configuration followed Petrova et al. [31], and the craniofacial configuration was based on the protocol described by Paoloni et al. [23]; all manual landmark placement and cephalometric tracings were performed by a single examiner (MS).

### 2.8. Bias

Several steps were taken to reduce potential bias. To limit selection bias, only subjects with complete documentation and adequate-quality maxillary casts and lateral cephalometric radiographs were included, although selection bias related to the availability and quality of archived records could not be fully eliminated. To reduce measurement bias, palatal and craniofacial morphology were assessed using predefined geometric morphometric protocols; all casts were digitized on the same scanner, models were landmarked using the same protocol, and right-sided clefts were mirrored so that the cleft side was consistently represented on the left. All landmarks were placed by a single examiner. Age was not included in the primary PLS analysis.

All landmarking was performed on the digital palatal models or digital cephalometric tracings. Landmark-placement repeatability was evaluated separately for palatal models and lateral cephalograms by repeating the complete landmarking procedure on a random subset of 25 maxillary models and 25 cephalograms after an interval of at least one month. Error was assessed from the Procrustes distance between replicate shape configurations and expressed relative to the total shape variance of the corresponding sample. This was a repeat-digitization distance approach rather than a Procrustes ANOVA.

### 2.9. Sample Size

No formal a priori sample-size calculation was performed because this retrospective study was based on a fixed archival cohort. All patients who fulfilled the predefined eligibility criteria and had complete clinical records, adequate maxillary dental casts, and adequate lateral cephalometric radiographs were included. A conventional post hoc observed-power calculation was not used because it would largely re-express the observed *p*-value and would not appropriately represent the high-dimensional permutation-based two-block PLS/RV analysis. The modest sample size was therefore retained as a limitation.

### 2.10. Statistical Methods

Landmark configurations were subjected to Generalized Procrustes Analysis (GPA) [33] to remove the effects of translation, rotation, and scale; configurations were processed in Viewbox 4 (dHAL Software, Kifissia, Greece), and subsequent analyses were performed in MorphoJ (version 1.08.02). Principal component analysis (PCA) was carried out separately for the palatal and craniofacial datasets to summarize the main axes of shape variation within each block. Sex-related differences in palatal and craniofacial shape were assessed by permutation tests (10,000 permutations) using the Procrustes distance between group means as the test statistic; because no statistically significant differences were detected, both sexes were pooled. Covariation between palatal and craniofacial shape was assessed using two-block partial least squares (PLS) analysis [34]. The global association was quantified using Escoufier’s RV coefficient [35], with statistical significance evaluated by 10,000 permutations. As an independent validation, the subject pairing between the blocks was additionally randomized 100,000 times to obtain a design-specific permutation-null distribution of RV. PLS1 reproducibility was assessed in 5000 paired bootstrap resamples. Bootstrap modes were matched to the full-sample PLS1 while accounting for sign changes and changes in PLS mode order. A 10,000-permutation analysis provided a reference for the observed directional changes. Bootstrap percentile ranges were also examined for the matched-mode block-score correlation and raw RV; full details are provided in Figure S3. Confidence intervals and percentile ranges were used to summarize estimation uncertainty [36]. As an additional sensitivity analysis, age-related variation was removed separately from the palatal and craniofacial Procrustes coordinates by linear regression on age at dental-cast acquisition, after which the two-block PLS/RV analysis was repeated using 10,000 permutations. No imputation of missing data was performed. Statistical significance was set at *p* < 0.05. Permutation tests do not require inversion of a large covariance matrix and remain applicable when the number of coordinate variables exceeds the number of patients; nevertheless, a modest sample can reduce sensitivity to subtle associations and make data-derived PLS patterns less reproducible.

## 3. Results

### 3.1. Study Sample

**Figure 3:**
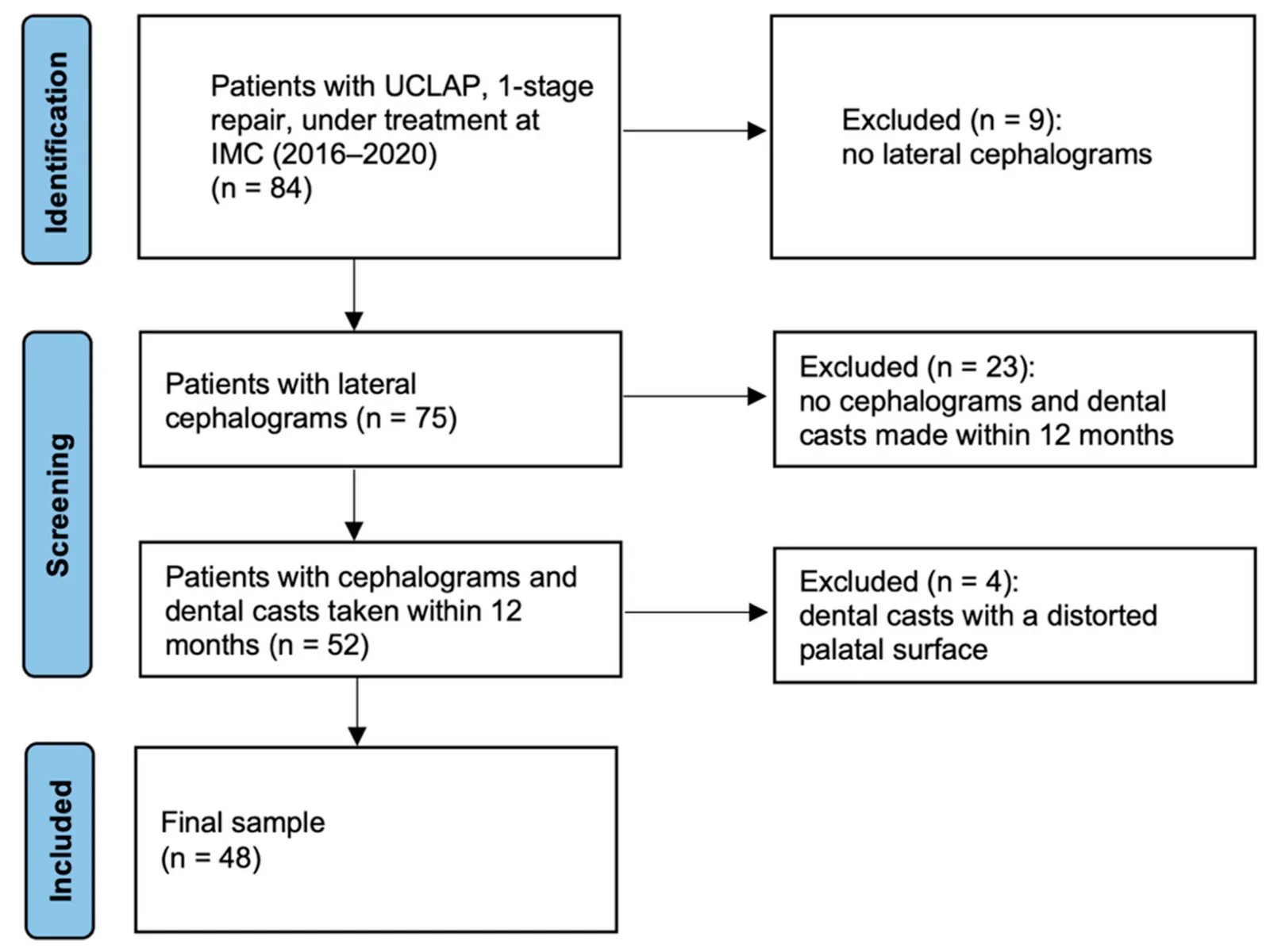
Flow diagram of participant screening, exclusions, and inclusion in the final study sample. IMC, Institute of Mother and Child; UCLAP, unilateral cleft lip, alveolus, and palate.

Participant screening and selection are presented in the flow diagram (Figure 3). At the final screening step, four patients were excluded because their dental casts had a distorted palatal surface. The final study sample comprised 48 participants (33 boys and 15 girls). The mean age at maxillary cast acquisition was 9.86 years (SD 0.89; median 9.9; IQR 9.4–10.1; range 8.0–13.6 years). The mean within-patient difference in acquisition age between the lateral cephalogram and dental cast was −0.02 ± 0.69 years; the median difference was 0.00 years (IQR −0.80 to 0.25), with a range from −1.00 to 1.00 years. Palatal and craniofacial shape did not differ significantly between girls and boys (10,000 permutations; palatal *p* = 0.74; craniofacial *p* = 0.25); therefore, both sexes were combined in subsequent analyses (Figures S1 and S2).

### 3.2. Digitization Error

The mean random error of the 25 repeated digitizations, expressed as a percentage of total shape variance, was 4.8% and 5.9% for maxillary casts and lateral cephalograms, respectively.

### 3.3. Procrustes Superimposition and PCA

**Figure 4:**
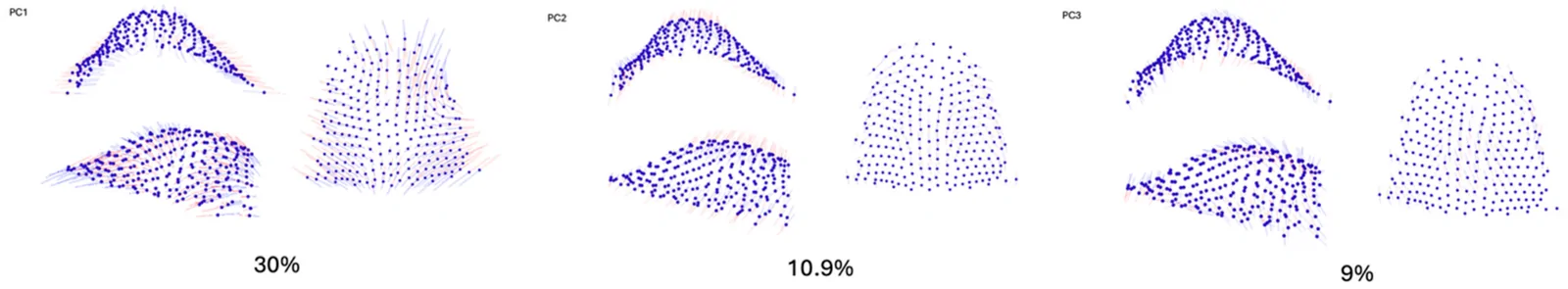
Shape changes along the first three principal components (PCs) of palatal morphology, shown in frontal, oblique, and occlusal views. Red represents the configuration at −3 standard deviations and blue the configuration at +3 standard deviations from the mean along each PC; thus, the illustrated change spans 6 standard deviations. Shape change should be read from red (negative PC scores) toward blue (positive PC scores). Greater separation between corresponding landmarks indicates a larger local shape change. Percentages indicate the proportion of total palatal shape variance explained by each PC.

For palatal morphology, the initial twenty-two principal components (PCs) captured non-trivial variation in shape, collectively accounting for 92.5% of the overall shape variability. The first six PCs each contributed at least 5% of the total shape variability: PC1 = 30%, PC2 = 10.9%, PC3 = 9%, PC4 = 7.9%, PC5 = 7.2%, and PC6 = 5% (69.9% in total) (Table S1). PC1 primarily characterized morphological variation in palatal width and length, displaying noticeable patterns of wide-and-short versus narrow-and-long; PC1 also revealed variation related to the cleft region. PC2 predominantly reflected variation in palatal height, whereas PC3 indicated cleft side-related variation across all three dimensions (Figure 4).

**Figure 5:**
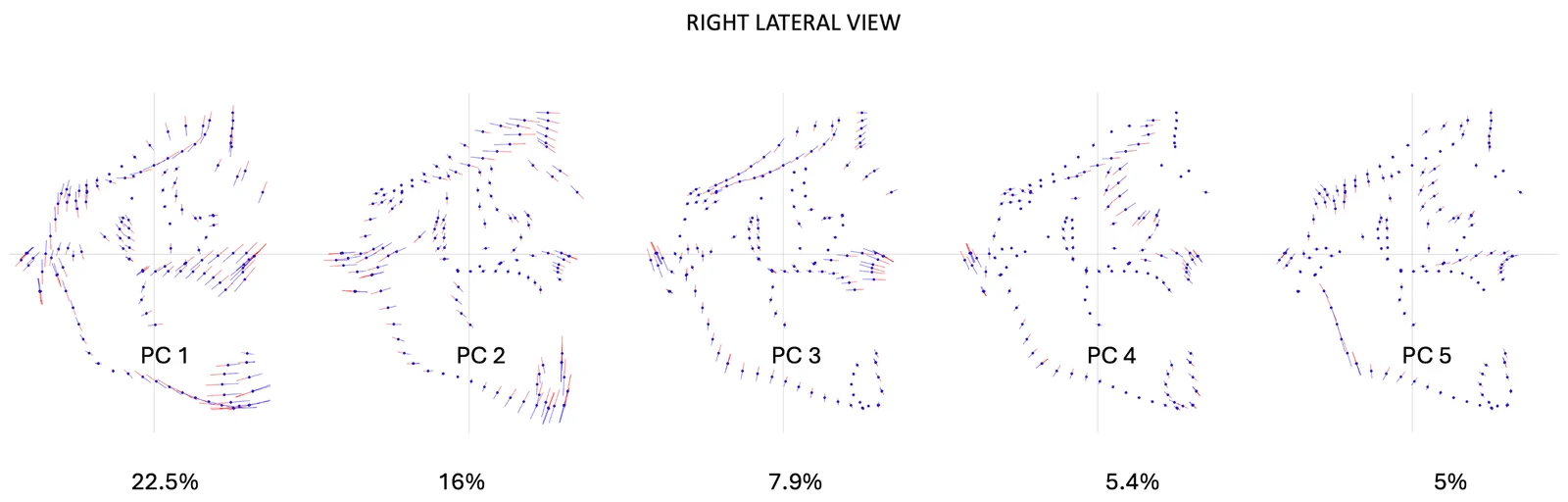
Shape changes along the first five principal components (PCs) of craniofacial morphology, shown in right lateral view. Red represents the configuration at −3 standard deviations and blue the configuration at +3 standard deviations from the mean along each PC; thus, the illustrated change spans 6 standard deviations. Shape change should be read from red (negative PC scores) toward blue (positive PC scores). Greater separation between corresponding landmarks indicates a larger local shape change. Percentages indicate the proportion of total craniofacial shape variance explained by each PC.

For craniofacial morphology, the first five principal components accounted for 56.8% of the total shape variance (Table S2). PC1 explained the largest proportion of variance (22.5%) and mainly reflected anteroposterior variation in the mandibular region, with a secondary contribution from anteroposterior variation in the maxillary region. PC2 explained 16.0% of the total variance and was primarily associated with vertical variation in the mandibular dentoalveolar segment. The remaining three components—PC3, PC4, and PC5—accounted for 7.9%, 5.4%, and 5% of the total variance, respectively (Figure 5).

### 3.4. Two-Block Partial Least Squares Analysis

**Figure 6:**
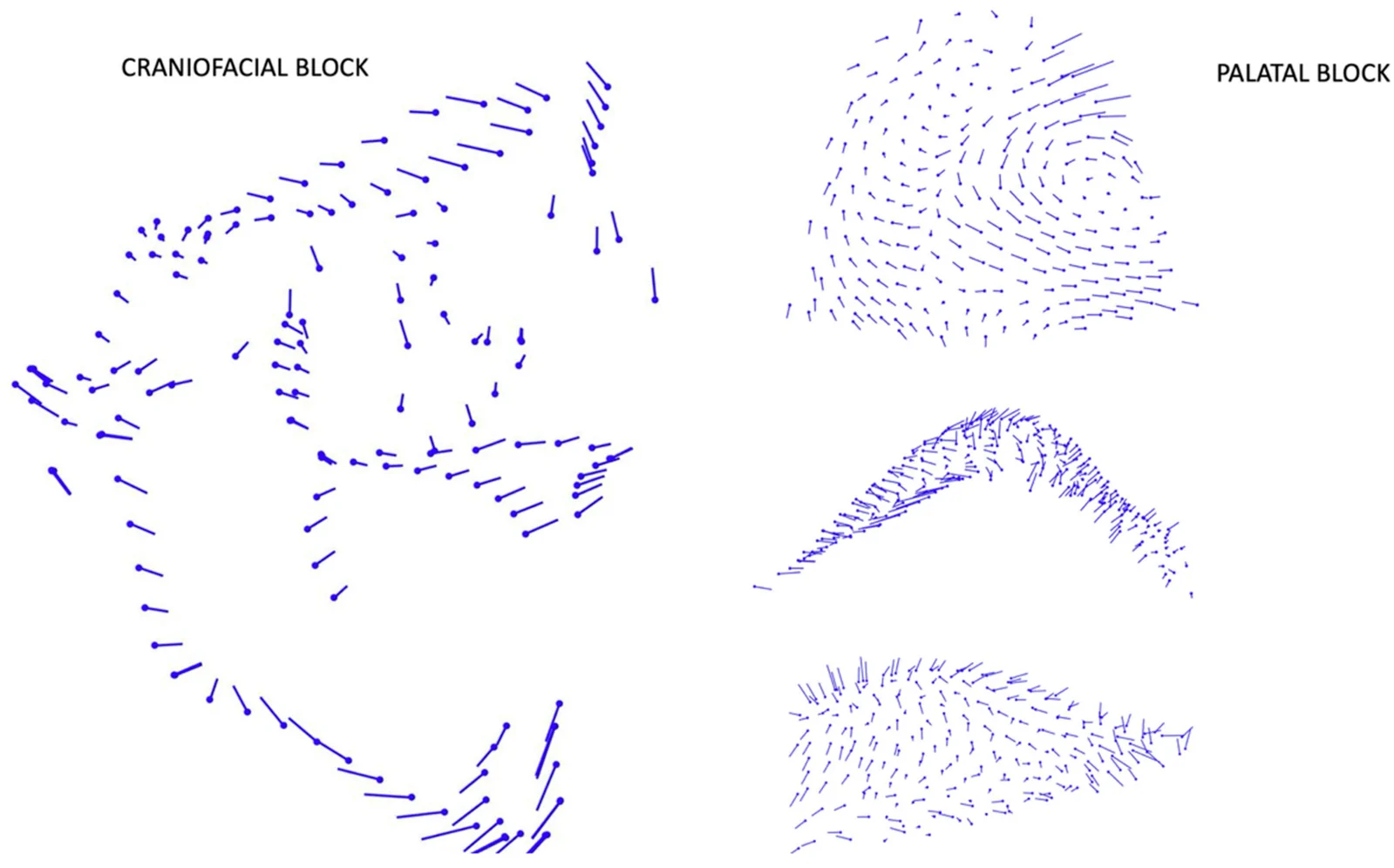
Shape changes along the first pair of two-block partial least squares axes (PLS1), shown for the craniofacial block on the left and the palatal block on the right. Vectors indicate the direction of landmark displacement along PLS1, and vector length represents the relative magnitude of displacement; longer vectors indicate greater local shape change. Because PLS1 was not statistically significant and showed only partial bootstrap reproducibility, the illustrated anatomical pattern should be interpreted as exploratory.

Two-block partial least squares analysis did not show statistically significant overall covariation between palatal and craniofacial morphology (RV = 0.254, *p* = 0.097; 10,000 permutations). The first PLS mode accounted for 28.9% of the total squared covariance and showed a block-score correlation of r = 0.658, which was also not statistically significant (*p* = 0.084) (Figure 6).

Bootstrap resampling showed only partial reproducibility of the PLS1 pattern. The direction of PLS1 was more consistent than expected under random pairing, but substantial variation remained across resamples. The relatively high bootstrap block-score correlations did not indicate a stable anatomical association because PLS recalculates the axes in each resample to maximize covariance. Detailed bootstrap results are provided in Figure S3 and Supplementary Analysis S1.

### 3.5. Age-Adjusted Sensitivity Analysis

Adjusting for age did not meaningfully change the results. The age-adjusted analysis showed RV = 0.261 (*p* = 0.091) and a PLS1 block-score correlation of r = 0.653 (*p* = 0.118), with PLS1 accounting for 31.4% of total squared covariance. This suggests that age differences within the study group did not substantially influence the main findings.

### 3.6. Permutation Validation of the Global RV Test

An independent 100,000-permutation analysis was performed to confirm the primary RV result. The mean RV expected under random pairing of palatal and craniofacial data was 0.223, and the 95th percentile was 0.264. The observed RV of 0.254 remained below this threshold, with *p* = 0.095, closely reproducing the original MorphoJ result (*p* = 0.097).

Because the bootstrap distribution of raw RV was shifted upward relative to the full-sample estimate, it was not treated as a conventional confidence interval for the population RV. The permutation analysis was therefore used as the main basis for inference on the overall association (Figure S3).

## 4. Discussion

The present study explored covariation between palatal shape and craniofacial morphology in children with UCLAP using geometric morphometric methods. We found no clearly defined or statistically significant pattern of shared shape variation between the two blocks: the global RV test was nonsignificant, and the first PLS mode was also nonsignificant. In addition, bootstrap resampling showed substantial directional variability and only partial reproducibility of the PLS1 pattern. The anatomical changes represented by PLS1 should therefore be regarded as exploratory and specific to this sample rather than as evidence of a reproducible pattern of morphological integration. Taken together, these findings suggest that palatal shape alone may not reliably reflect the overall craniofacial configuration.

The two morphological blocks differed in dimensional representation. The palate was assessed three-dimensionally, whereas the craniofacial complex was represented by two-dimensional lateral cephalograms. Asymmetry and transverse discrepancies are central components of UCLAP morphology but are poorly represented on lateral cephalograms. Consequently, potentially relevant shared transverse or asymmetric variation may not have been captured adequately, which could have reduced the ability of the analysis to identify a stable cross-block association. Fully three-dimensional craniofacial assessment would allow this issue to be tested directly in future studies.

The dominant axes of craniofacial variation observed here resemble those reported in untreated cleft cohorts, in which variability is expressed more strongly in the anteroposterior than in the vertical direction, for both unilateral [27] and bilateral clefts [37]. This contrasts with non-cleft studies, in which palate–craniofacial covariation has commonly been associated with the vertical dimension, with high-angle patterns related to a narrower and higher palate [22,23,24,26,38]. Previous morphometric studies in non-cleft Class II and Class III samples have also described relatively concentrated covariation on the first PLS axis [22,23]. However, direct numerical comparison of raw RV coefficients across studies is not appropriate because the expected RV depends on sample size, dimensionality, and covariance structure. In the present UCLAP sample, PLS1 accounted for 28.9% of total squared covariance, but this axis was not statistically significant. It should therefore be regarded as a descriptive observation rather than evidence of a reproducible integration pattern.

Although the PLS1 block-score correlation was relatively high numerically (r = 0.658), it should not be interpreted using conventional thresholds proposed for Pearson correlations between predefined variables. In PLS, the score axes are derived specifically to maximize covariance between the two morphological blocks, and the resulting correlation therefore has a different statistical meaning from an ordinary Pearson correlation. Its magnitude should consequently be considered together with the permutation results and the directional reproducibility of PLS1 rather than interpreted in isolation.

Previous studies provide a biological context for the considerable morphological variability observed in patients with clefts. Geometric morphometric studies of untreated clefts have described differences in craniofacial integration and modularity [27,39,40], whereas operated UCLAP patients have been reported to show broader craniofacial variability than unaffected individuals [28]. These findings suggest that cleft-related craniofacial development may differ from that of non-cleft populations. However, because the present study did not include a matched non-cleft control group, it cannot determine whether clefting or its treatment alters a normal pattern of palate–craniofacial integration.

Surgical treatment is integral to UCLAP management, and palatal surgery may influence subsequent maxillary and palatal morphology. Trials and systematic reviews have not identified a clearly superior primary repair protocol with respect to maxillary growth or dental arch relationships [41,42,43,44,45]. Palatal scarring and wound contraction have also been discussed as possible contributors to altered maxillary growth [46,47], and palate repair has been associated with impaired sagittal maxillary development in some studies [7,48,49]. These findings provide relevant clinical context, but the present study was not designed to determine the individual contribution of surgery to the observed morphology.

Dental anomalies may provide an additional source of morphological heterogeneity. Tooth agenesis is common in non-syndromic clefts and may occur outside the cleft region [50,51,52]. Tooth number has also been associated with craniofacial morphology [53]. Because dental anomalies were not analyzed separately in the present study, their possible influence should be regarded as a potential source of heterogeneity rather than as an explanation for the observed results.

Orthodontic treatment may also contribute because therapeutic changes to the maxilla and dental arches are superimposed on intrinsic growth and cleft-related development. Maxillary expansion can increase arch width and perimeter and modify palatal and dental-arch morphology [54,55], while more extensive orthodontic or orthopedic treatment may also affect dentoskeletal relationships [56,57,58]. Because patient-level treatment detail was not consistently available (see Section 2), treatment exposure could not be quantified or adjusted for, and residual treatment-related effects on palatal morphology cannot be excluded. In addition, baseline cleft severity represents another potential source of heterogeneity. As initial cleft dimensions may influence subsequent palatal and maxillary development, their possible contribution could not be assessed or controlled for in the present study.

A strength of the present study is the direct two-block assessment of palatal–craniofacial covariation in operated complete UCLAP, whereas previous cleft studies have generally evaluated craniofacial and palatal morphology separately. Additional strengths include dense three-dimensional sampling of palatal shape, paired dental-cast and cephalometric records obtained within 12 months, a single-center cohort with a relatively standardized surgical protocol, and permutation and bootstrap procedures that assessed both statistical evidence and the reproducibility of the data-derived PLS pattern. These features provide a more direct test of whether palatal morphology reflects the overall craniofacial configuration in this population.

These findings should be interpreted within the context of a single-center cohort with a relatively homogeneous surgical protocol. The results therefore apply primarily to comparable non-syndromic complete UCLAP populations and should not be generalized directly to bilateral, isolated, syndromic, untreated, or differently treated clefts. The retrospective cross-sectional design also allows assessment of morphological association at one developmental period but cannot establish the direction of the relationship or separate the effects of congenital morphology, growth, surgery, alveolar bone grafting, and orthodontic treatment. Several additional limitations should be acknowledged. The sample size was modest relative to the complexity of the geometric morphometric data and may have limited the detection of weak or heterogeneous associations and the stability of data-derived PLS directions. Detailed patient-level orthodontic treatment information (including dentition stage, timing, duration, and appliance exposure) and quantitative baseline cleft-severity measurements were unavailable. Age was not included in the primary PLS analysis; however, an age-residualized sensitivity analysis produced similar RV and PLS1 results, reducing concern that the observed findings were driven by age-related shape variation. All paired dental casts and lateral cephalograms were obtained within 12 months of each other, limiting major temporal mismatch, although developmental changes within that interval cannot be excluded. Palatal morphology was reconstructed from plaster casts that were subsequently optically digitized. Although TRIOS-based digitization of gypsum models has been evaluated for orthodontic model measurements [30], possible errors related to cast production and scanning were not quantified separately by the repeat-landmarking assessment. No matched non-grafted comparison group with both palatal and cephalometric records was available, and the present dataset was not designed to support a subgroup comparison according to alveolar bone-grafting timing. This is particularly relevant because patients in the present cohort underwent early secondary alveolar bone grafting most often between 2 and 4 years of age, whereas graft timing varies among treatment protocols. Consequently, the present study cannot determine whether the observed morphology or the absence of a stable palate-craniofacial association would differ in patients treated at other grafting ages, nor can graft-related effects be reliably separated from cleft-related development and the effects of other treatment stages. Finally, no matched non-cleft control group with both palatal and craniofacial data was available. These limitations require cautious interpretation of the nonsignificant global association and the exploratory PLS1 anatomy. Future studies specifically designed to compare non-grafted patients and/or groups treated according to different graft-timing protocols could help distinguish treatment-related from developmental variation.

## 5. Conclusions

In children with operated complete unilateral cleft lip, alveolus, and palate, palatal shape did not prove to be a dependable marker of craniofacial morphology, and these regions are therefore better assessed directly than inferred from one another. Future studies should use fully three-dimensional imaging of both structures, longitudinal follow-up to disentangle growth from treatment effects, and larger multicentre samples with recorded cleft severity and treatment history, so as to determine whether clinically relevant palate–craniofacial relationships can be established.

## Data Availability

The data presented in this study are available on request from the corresponding author. The data are not publicly available owing to privacy and ethical restrictions.

## Supplementary Materials

The following supporting information can be downloaded at: https://www.mdpi.com/article/10.3390/jcm15186939/s1, Table S1: Variance explained by each non-trivial principal component (PC) of palatal shape; Table S2: Variance explained by each non-trivial principal component (PC) of craniofacial shape; Figure S1: Distribution of boys and girls in craniofacial PCA shape space; Figure S2: Distribution of boys and girls in palatal PCA shape space; Figure S3: Design-specific permutation null distribution of the global RV coefficient; Supplementary Analysis S1: PLS1 bootstrap reproducibility; Supplementary Analysis S2: Age-adjusted sensitivity analysis.
