# Supplementary material for: Covariation Between Palatal Shape and Craniofacial Morphology in Complete Unilateral Cleft Lip, Alveolus, and Palate: A Geometric Morphometric Study
Source: J Clin Med. 2026 Sep 8;15(18):6939. doi: 10.3390/jcm15186939 (PMC13607854; doi:10.3390/jcm15186939)
Supplement: Supplementary file 1 [file jcm-15-06939-s001.zip › jcm-4506025-supplementary.pdf]

Supplementary Material

Table S1. Variance explained by each non-trivial principal component (PC) of palatal shape. For each PC, the percentage of total shape variance, the cumulative percentage, and the corresponding broken-stick criterion are given.

PC	% variance	% cumulative variance	broken-stick criterion
PC 1	30.0%	30.0%	1.0%
PC 2	10.9%	40.8%	0.9%
PC 3	9.0%	49.8%	0.8%
PC 4	7.9%	57.7%	0.7%
PC 5	7.2%	64.9%	0.7%
PC 6	5.0%	69.9%	0.7%
PC 7	3.9%	73.8%	0.7%
PC 8	2.7%	76.5%	0.6%
PC 9	2.3%	78.9%	0.6%
PC 10	2.0%	80.8%	0.6%
PC 11	1.8%	82.6%	0.6%
PC 12	1.4%	84.0%	0.6%
PC 13	1.3%	85.3%	0.6%
PC 14	1.2%	86.5%	0.6%
PC 15	1.1%	87.6%	0.5%
PC 16	1.0%	88.6%	0.5%
PC 17	0.8%	89.4%	0.5%
PC 18	0.8%	90.2%	0.5%
PC 19	0.7%	90.9%	0.5%
PC 20	0.6%	91.4%	0.5%
PC 21	0.6%	92.0%	0.5%
PC 22	0.5%	92.5%	0.5%

Table S2. Variance explained by each non-trivial principal component (PC) of craniofacial shape. For each PC, the percentage of total shape variance, the cumulative percentage, and the corresponding broken-stick criterion are given.

PC	% variance	% cumulative variance	broken-stick criterion
PC 1	22.5%	22.5%	2.1%
PC 2	16.0%	38.5%	1.7%
PC 3	7.9%	46.4%	1.6%
PC 4	5.4%	51.8%	1.5%
PC 5	5.0%	56.8%	1.4%
PC 6	4.3%	61.1%	1.3%
PC 7	3.8%	64.9%	1.3%
PC 8	3.4%	68.3%	1.2%
PC 9	3.0%	71.2%	1.2%
PC 10	2.7%	73.9%	1.1%
PC 11	2.6%	76.5%	1.1%
PC 12	2.1%	78.7%	1.1%
PC 13	2.0%	80.7%	1.1%
PC 14	1.6%	82.3%	1.0%
PC 15	1.5%	83.8%	1.0%
PC 16	1.3%	85.1%	1.0%
PC 17	1.1%	86.2%	1.0%
PC 18	1.1%	87.3%	0.9%
PC 19	1.0%	88.3%	0.9%
PC 20	0.9%	89.2%	0.9%

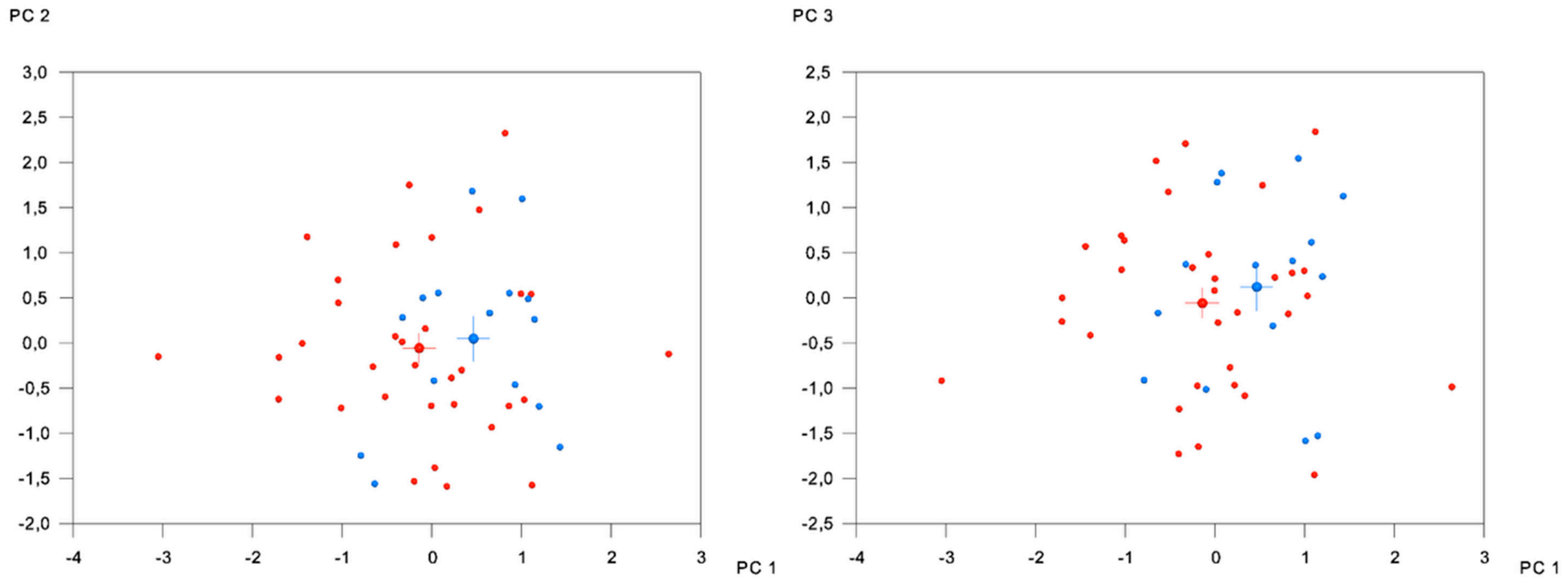

Figure S1. Distribution of boys (red circles) and girls (blue circles) in the shape space described by principal components 1 and 2 (PC1 and PC2) and principal components 1 and 3 (PC1 and PC3) for craniofacial morphology

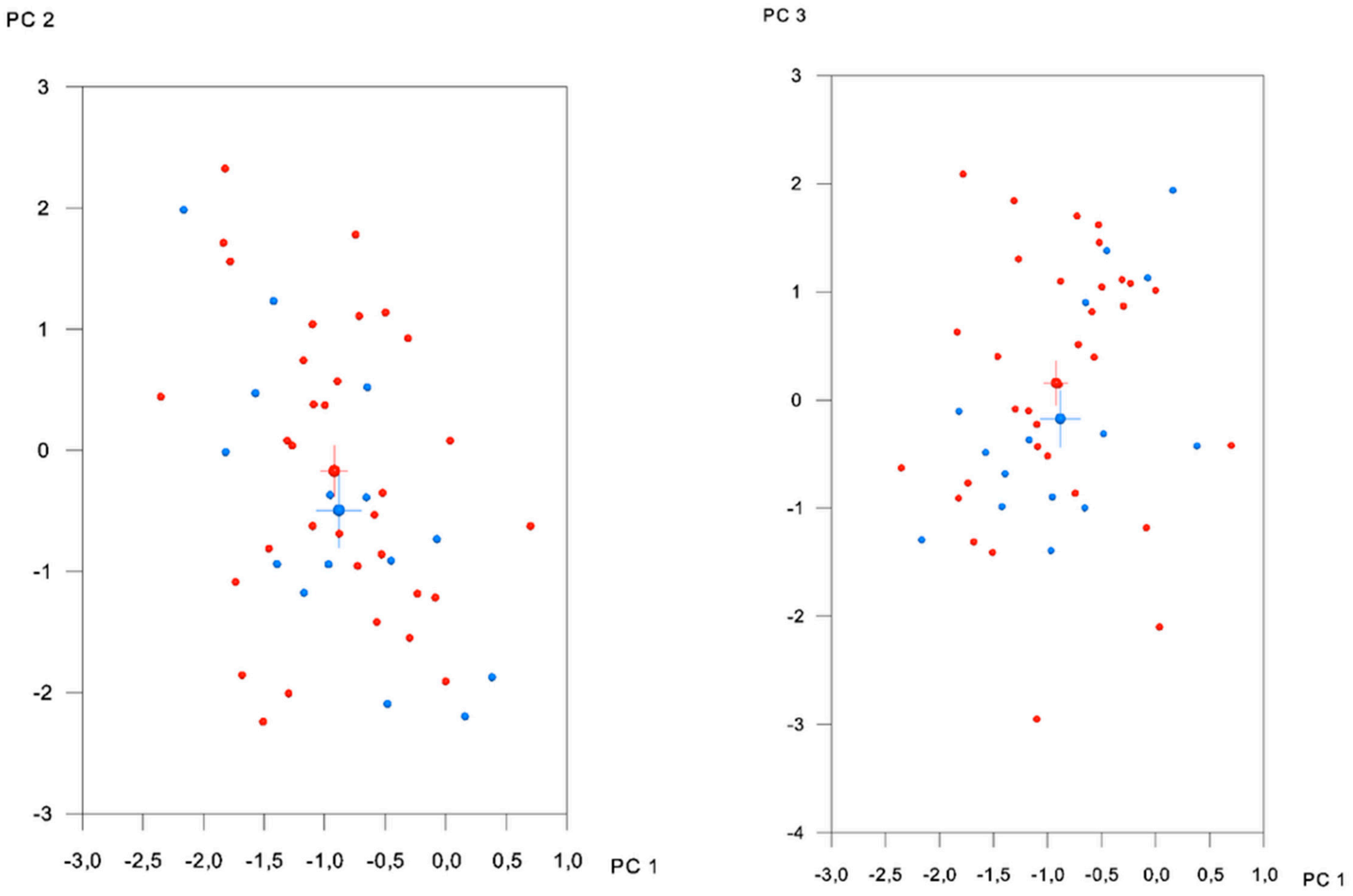

Figure S2. Distribution of boys (red circles) and girls (blue circles) in the shape space described by principal components 1 and 2 (PC1 and PC2) and principal components 1 and 3 (PC1 and PC3) for palatal shape

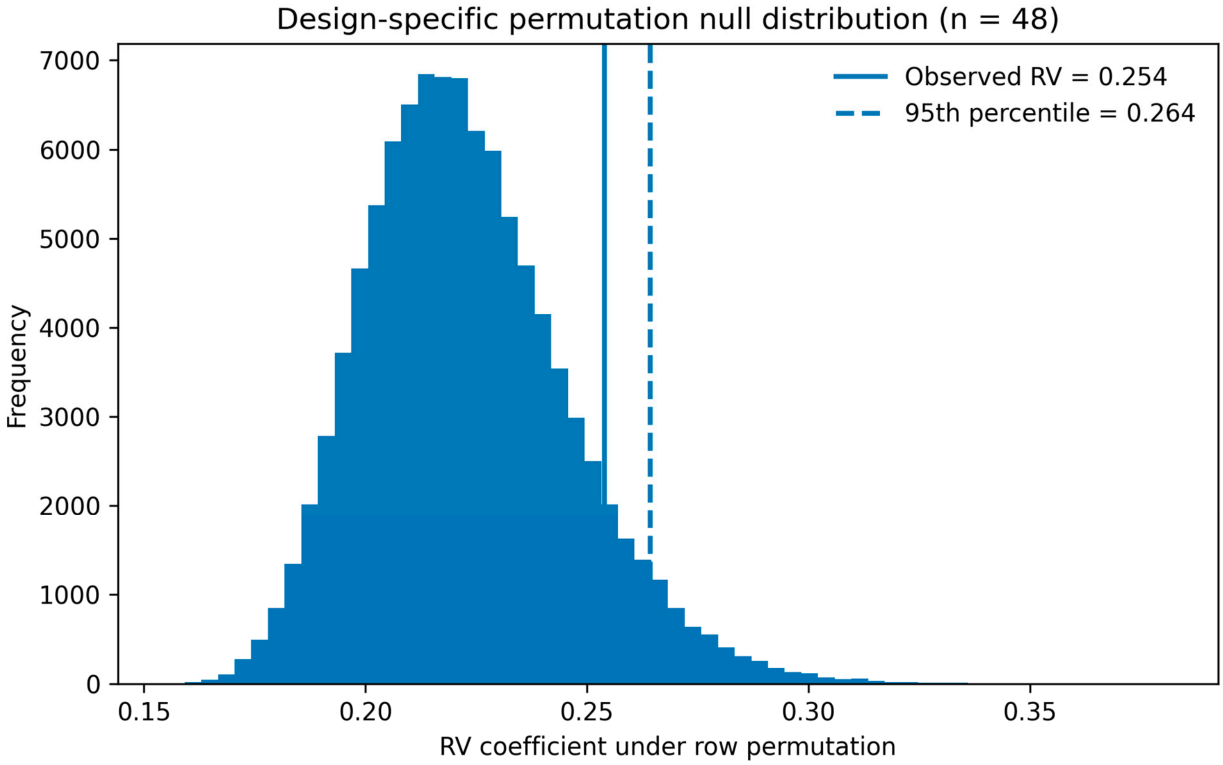

Figure S3. Design-specific permutation null distribution of the global RV coefficient based on 100,000 random row permutations for n = 48. The observed RV = 0.254 and the 95th percentile of the permutation null = 0.264 are indicated.

Palatal (239 three-dimensional points) and craniofacial (152 two-dimensional points) configurations were subjected separately to Generalized Procrustes Analysis. The global RV coefficient and first two-block PLS mode were recalculated. The finite-sample null distribution of RV was estimated by 100,000 random row permutations of the craniofacial block while holding the palatal block fixed. PLS1 directional reproducibility was assessed in 5,000 paired bootstrap resamples. To account for sign ambiguity and changes in PLS mode order, bootstrap modes were matched to the full-sample PLS1 by maximizing sign-invariant directional congruence across the palatal and craniofacial blocks before acute angular deviation was calculated. A 10,000-permutation angular reference distribution was generated using the same matching rule. The matched-mode block-score correlation was also recorded.

The re-analysis reproduced the manuscript results: RV = 0.254, PLS1 score correlation r = 0.658, and PLS1 = 28.9% of total squared covariance. The 100,000-permutation validation yielded p = 0.095, closely matching the MorphoJ result (p = 0.097). The permutation-null mean RV was 0.223 and its 95th percentile was 0.264. Thus, the observed RV remained below the 95th percentile.

Supplementary Analysis S1. PLS1 Bootstrap Reproducibility

After matching each bootstrap PLS pattern to the pattern most similar to the original PLS1, the median change in direction was 45.7° for the palatal block and 42.1° for the craniofacial block (central 95% ranges: 25.8°–65.2° and 22.3°–63.0°, respectively). These changes were smaller than those obtained when the two blocks were paired at random (median 62.0° and 56.7°; central 95% ranges: 38.9°–80.6° and 33.1°–79.8°, respectively). The corresponding pattern was identified as PLS1 in 52.8% of bootstrap samples, as PLS2 in 34.6%, and as a higher-order mode in 12.6%, indicating frequent changes in PLS mode order. Overall, the results indicate some consistency in direction but only partial reproducibility of the PLS1 pattern. The matched block-score correlation had a 95% bootstrap percentile range of 0.634–0.850. Because PLS recalculates its axes in each resample to

maximize covariance, this relatively high correlation should not be interpreted as evidence that the same anatomical association was reproduced.

Supplementary Analysis S2. Age-Adjusted Sensitivity Analysis

Linear age-related variation was removed separately from the palatal and craniofacial Procrustes coordinates by regression on age at dental-cast acquisition, and the two-block analysis was repeated using 10,000 permutations. The age-residualized results were RV = 0.261 ($p = 0.091$), PLS1 block-score correlation $r = 0.653$ ($p = 0.118$), and PLS1 = 31.4% of total squared covariance. These findings were similar to the primary analysis.
